# Oral Lesions and Dental Status in Individuals Using Oral Tobacco‐Free Nicotine Pouches

**DOI:** 10.1111/odi.70409

**Published:** 2026-07-01

**Authors:** Masoumeh Daneshian, Lars Fredriksson, Pia Skott, Karin Garming Legert

**Affiliations:** ^1^ Public Dental Services Stockholm County Council Stockholm Sweden; ^2^ Department of Dental Medicine Karolinska Institutet Stockholm Sweden

**Keywords:** nicotine pouches, oral lesions, smokeless tobacco, snus‐induced lesions, Swedish oral tobacco‐derived snus, Swedish snuff

## Abstract

**Objectives:**

Users of Swedish nicotine pouches and traditional oral tobacco‐derived pouches were compared to non‐users for clinical tissue responses and oral health status. In addition, the habits associated with snus use were also evaluated.

**Material and Methods:**

In this cross‐sectional study, participants aged 18–30 were recruited from nine dental clinics: 126 nicotine pouch users, 63 oral tobacco‐derived pouch users, and 83 non‐users. Clinical examinations assessed mucosal changes and dental status, including erythema, ulceration, oedema, hyperkeratosis, etching, bone defects, and gingival recession. Participants completed a questionnaire on snus use, oral hygiene, and education. Tissue responses were graded and intergroup differences analysed. The study followed the STROBE checklist.

**Results:**

Oral lesions were identified in 79% of nicotine pouch users and 89% of oral tobacco‐derived pouch users. Nicotine pouch users more frequently exhibited erythema and reported more oral symptoms, while hyperkeratosis predominated among oral tobacco‐derived pouch users. A non‐significant trend suggested mint‐flavoured nicotine pouches may be associated with more pronounced lesions. No increased risk of caries or periodontal disease was observed for ≤ 6 years of use.

**Conclusions:**

Nicotine pouches appear to induce broader mucosal responses than tobacco‐containing snus. Further research is needed to clarify local and systemic effects.

## 1 Introduction

It is well known that the use of tobacco and nicotine products involves general systemic health risks and is associated with poorer oral health. Risk varies depending on the product, with cigarettes being particularly associated with an increased risk of periodontitis, gingivitis, caries, and oral cancer (Chaffee et al. 2021; Hajat et al. 2021; Warnakulasuriya and Muthukrishnan 2018). Similar reports occur for Swedish moist snus, technically classified as oral tobacco‐derived (OTD) snus, concerning general systemic health effects. Unlike smoking, however, epidemiological studies have been unable to demonstrate any correlation between OTD snus and oral diseases, such as caries and periodontitis (Rolandsson et al. 2005; Hugoson and Rolandsson 2011; Hugoson et al. 2012; Hellqvist et al. 2015). Moreover, several studies have been unable to find an association between snus use and oral and pharyngeal cancer (Lewin et al. 1998; Araghi et al. 2021). Results from several meta‐analyses support this conclusion (Boffetta et al. 2008; Lee 2011; Lee and Hamling 2009).

The term ‘snus’ encompasses a wide range of products, from dry snus for inhalation to various types of moist snus. It is well documented that the levels of carcinogenic nitrosamines in tobacco snus vary significantly between countries due to differences in tobacco processing and storage conditions. The International Agency for Research on Cancer (International Agency for Research on Cancer 2007) has therefore classified snus as a risk factor for oral cancer.

Snus has a long history in Sweden, and today, Swedish snus has become deeply embedded into Swedish culture and public health. Snus was first introduced in the early 17th century and evolved during the 18th and 19th centuries into the moist oral preparation now associated with Swedish OTD snus. In the mid‐1970s, snus portions were introduced in Sweden, and today, OTD snus products are sold either as loose snus or as pouches. In 2016, nicotine pouches (NPs) were introduced on the Swedish market. The content of NPs varies slightly between manufacturers, but they generally contain flavouring agents, sweeteners, pH adjusters, and fillers, such as plant‐based fibres or cellulose, with the purified nicotine that is either synthetically produced or extracted from tobacco (Forte 2025). It is not possible to determine the exact composition contained in each pouch based on the limited information provided by the manufacturers in the ingredient list.

NPs do not include tobacco leaves or fibres and are therefore frequently marketed as tobacco free. NPs deliver nicotine and maintain an alkaline pH similar to traditional OTD snus. However, NPs contain markedly lower or non‐quantifiable levels of tobacco‐specific or other harmful constituents, such as tar, lead, carbon monoxide, cadmium, hydrogen cyanide, and arsenic (Azzopardi et al. 2021). Nicotine is added separately, resulting in substantial variability in nicotine concentrations, and several widely consumed variants exhibit significantly higher nicotine concentrations compared to conventional tobacco products (Mallock et al. 2022). Furthermore, compared to conventional OTD pouches containing 8 mg of nicotine, NPs containing 6 mg of nicotine have been shown to result in a significantly greater systemic nicotine exposure, as measured by area under the plasma concentration time curve from time zero to infinity (AUC_inf_) (Lunell et al. 2020).

A characteristic feature of NPs is that they are sold in many different flavours than OTD snus. The wide range of flavours, fragrances, and all‐white appearance of the pouch, in contrast to traditional, brown‐coloured OTD pouches, is believed to be a substantial contributing factor to its popularity (Dowd et al. 2023). In addition to OTD snus and NPs, nicotine‐ and tobacco‐free snus substitute pouches, consisting mainly of flavoured plant fibres, are also available.

In Sweden in 2024, 22% of men and 10% of women aged 16–84 reported using snus daily. Usage has increased in recent years, especially among individuals aged 16–29. This increase can primarily be attributed to the growing use of NPs, particularly among young individuals. Among young Swedish women, daily use of NPs rose from 3% to 18% between 2018 and 2024, and among young men, from 5% to 11% between 2022 and 2024. In 2024, the prevalence of daily OTD snus use within the same age group was 5% among women and 18% among men, whereas the prevalence of daily smoking was 2% for both genders (Public Health Agency of Sweden 2025).

Considering that NPs were only recently launched in Sweden, the market has grown significantly in popularity. Sweden has been exempted from EU regulations regarding OTD snus since 1994, when the country joined the EU. This exemption does not apply to NPs. It is essential to acknowledge that the composition of NPs varies across countries due to substantial differences in national regulatory frameworks, including variations on how nicotine sources, additives, and product classifications are regulated (Duren et al. 2023). The European Commission has, however, expressed concern regarding their growing popularity, particularly among young people, and is therefore considering regulation or a potential ban. Several EU member states have already prohibited the sale of NPs, while others have introduced stricter regulations or are in the process of doing so. In the United States, NPs have become the subject of both Food and Drug Administration (FDA) marketing authorisation and political debate.

The rapid increase of NP use has raised public health concerns, as their clinical effects and impact on the oral mucosa remain insufficiently characterised. In contrast to the well‐documented clinical and histological alterations associated with long‐term use of OTD snus, the existing evidence regarding NPs is limited and fragmented. This scarcity of robust comparative data represents a substantial knowledge gap. Moreover, because NPs have only recently entered the market, prospective epidemiological studies on tissue reactions, general oral health outcomes, and potential cancer risk are lacking.

Snus is typically used by placing it under the upper lip against the gum, where the released substances are absorbed by the oral mucosa before entering the bloodstream. Snus‐induced mucosal lesions are commonly observed at the exact site of snus placement, with more pronounced manifestations associated with the use of loose snus compared to portioned pouches. In users of OTD snus, lesions have a characteristic clinical appearance: a well‐demarcated white or whitish‐grey patch on the oral mucosa where the surface is wrinkled, corrugated, or furrowed. In some cases, erythema may be visible, especially in new or irritated lesions (Axell et al. 1976). Gingival recession may also be present and result in exposure of dentine and root cementum, leading to tooth hypersensitivity and an increased susceptibility to dental caries (Kopperud et al. 2023).

If usage of OTD snus ceases, lesions will in most cases regress back to a completely normal mucosa within a few weeks. The gingiva, however, does not regenerate where it has retracted (Larsson et al. 1991). The pathophysiological mechanism behind snus‐induced lesions remains unclear. Histopathological findings of mucosal lesions associated with OTD snus include epithelial thickening, a vacuolated surface layer with eosinophilic, wave‐like projections, a narrow eosinophilic demarcation band, acanthosis, and mild subepithelial inflammation (Axell et al. 1976).

The high basic pH of snus, the effect of nicotine on the vascular bed, mechanical wear, and physical irritation have all been suggested as probable causes (Andersson and Warfvinge 2003; Joksić et al. 2019; Alizadehgharib et al. 2022). It is plausible that other components, such as moisture content of the snus pouches or the characteristics of the wrapping material, contribute to the development of snus‐induced mucosal lesions. Individual habits regarding snus usage, including brand, flavour, quantity, exposure time/pouch, whether the location of the snus pouch varies, and a dry mouth may simultaneously affect the occurrence, clinical appearance, and characteristics of snus lesions.

In users of NPs, our unpublished data from tissue biopsies reveal a spectrum of histopathological findings, ranging from changes similar to those observed in users of OTD snus to lichenoid inflammation accompanied by diffuse hyperkeratosis, occasionally with the presence of germinal centres. Germinal centres have been described in the oral tissue of patients sensitive to carvone, a mint flavour in spearmint oil used in, for example, toothpaste, which suggests that an immunological reaction has occurred (Kroona et al. 2018).

The limited evidence on NPs underscores the need for systematic investigations directly comparing their clinical and histopathological effects with those of OTD products under similar use conditions. Such comparisons are essential to determine whether the mucosal responses associated with NPs mirror, differ from, or exceed those induced by established OTD snus and to support evidence‐based risk assessment.

In this study, we assess the prevalence, clinical tissue response, and dental health in users of NPs and compare these findings with those observed in users of OTD snus and a group of non‐users. Additionally, we evaluate snus use habits in a cohort of young users in Sweden.

## 2 Materials and Methods

A question regarding daily snus consumption is a standard item in the Swedish dental health declaration, with four response categories: > ½, ½–1, 1–2, and > 2 cans of snus. Inclusion data for users of at least half a can of snus was obtained from all general dental clinics in the Public Dental Service of Stockholm County Council reporting 10% or more of their clientele registering daily consumption, regardless of type. Data on snus use were extracted from the patients' digital health declarations, submitted to their dental records up to 3 years prior by the Department of Research and Development. Nine of 54 clinics met these criteria and were evenly distributed across Stockholm County Council, representing a broad range of socioeconomic conditions. The staffing levels of the dental clinics ranged from five to eight dentists.

The Ethical Review Authority, Sweden approved the study (Etikprövningsmyndigheten, Uppsala, Sverige; Registration no. [Dnr] 2022‐04815‐01). The Declaration of Helsinki guidelines and the recommendations of the International Conference on Harmonisation—Good Clinical Practice (ICH‐GCP) were followed. The study is reported in accordance with the STROBE checklist.

### 2.1 Participants

Men and women who fulfilled the inclusion criteria were invited to participate during a routine dental visit at one of the selected clinics. Following signed informed consent, 272 participants were enrolled. Participants were classified into three groups according to their self‐reported snus type or non‐use: 126 users of NPs (62 men, 64 women), 63 users of OTD snus (57 men, 6 women), and 83 non‐users (35 men, 48 women). Participants were scheduled for a follow‐up appointment comprising a clinical examination and study questionnaires. Inclusion criteria were age 18–30 years, and daily use of five or more pouches (approximately a quarter of a can) for a minimum of 3 months and a maximum of 6 years. Since NPs had only been available on the market for 6 years at the time of the study, we chose this as the upper limit. For mixed users, nine out of 10 pouches consumed had to be the same type of pouch. Exclusion criteria were smoking, use of tobacco‐free snus substitute products (e.g., herbal smokeless tobacco pouches, caffeine pouches), use of other nicotine or tobacco products (excluding nicotine patches; these users were included), and a diagnosed oral disease or condition.

### 2.2 Clinical Examination

At each participating clinic, one main responsible dentist (the ‘research dentist’) was chosen to be trained according to the study protocol and be responsible for the clinical examination of the study participants. Training was conducted on site for 3 h at each participating clinic. Following a review of the study protocol, the research dentists practiced diagnosing changes in the oral mucosa using digitalised patient histories. Thus, nine research dentists were involved in data collection. Each research dentist chose participants based on the inclusion criteria, performed the clinical examination of the study participants from the clinic, and administered the questionnaire. The research dentists were also responsible for data collection and were available digitally or by telephone for inquiries from the study authors as needed. Each week, one of the study authors monitored data collection in an on‐site visit to one of the participating clinics; the clinics were monitored according to a rotating schedule.

The clinical examination of the oral mucosa focused on the identification and characterisation of oral mucosal changes at the habitual placement site of snus. When snus‐induced lesions were observed, clinical parameters, such as size and signs of erythema, ulceration, oedema, hyperkeratosis, etch damage, jawbone defect, and gingival recession, were recorded.

The dental examination comprised assessment of visible initial and manifest caries, periodontitis (periodontal probing depth [PPD] ≥ 4 mm in the incisal and molar regions and in the pouch area), presence of plaque and calculus (discrete, moderate, abundant), gingival recession (millimetre distance from the cementoenamel junction to the gingival margin), and gingival status (bleeding on probing [BoP]). When necessary, clinical findings were supplemented with pre‐existing radiographic data, particularly in cases where differentiation between snus‐induced gingival recession and periodontitis was required. Intraoral images of the oral mucosa were taken and entered in the patient's records in Planmeca Romexis, the clinic's x‐ray database.

### 2.3 Data Analysis and Clinical Examination

A clinical grading of the tissue response and severity of the snus‐induced lesion was done by the principal investigator (PI) (KGL) and one of the authors, based on photographs and the clinical examination protocol. Before grading, the examiners were calibrated against each other. A subset of 10 clinical photographs of diagnosed snus lesions was analysed in a blinded manner to ensure consistency in lesion classification prior to data interpretation. Using Cohen's kappa coefficient, inter‐rater reliability was assessed as 0.778 during calibration, indicating a substantial level of agreement (0.61–0.80).

To ensure a systematic and reproducible assessment, a standardised grading protocol was applied. Lesions in the NP group were evaluated by the PI and one of the authors using two novel grading systems developed specifically for this study to assess both tissue response and lesion severity. The grading scales were designed to capture the spectrum of tissue alterations typically associated with snus use, drawing on previously described clinical manifestations in OTD users and clinical observations in NP users, including erythema, oedema, ulceration, hyperkeratosis, and surface etching. Each lesion component was graded according to predefined criteria, allowing a nuanced and quantifiable description of the mucosal response and facilitating intergroup comparisons.

The *Tissue Reaction Scale* describes the tissue response in three grades: 0 = normal mucosa; 1 = predominantly hyperkeratosis; and 2 = predominantly erythema and/or ulceration. The *Severity Scale* classifies lesion severity in four grades: 0 = normal mucosa; 1 = mild (mild erythema and/or discrete hyperkeratosis); 2 = moderate (moderate erythema, ulceration, and/or hyperkeratosis); and 3 = severe (marked erythema, ulceration, and/or hyperkeratosis).

Snus‐induced lesions among users of OTD snus were graded using the four‐grade classification system of Axell et al. (1976). If a snus‐induced lesion in users of NPs was classified as grade 1 according to the Tissue Reaction Scale (predominantly hyperkeratosis), the four‐grade classification system of Axell et al. was also used to compare the prevalence and extent of the hyperkeratosis of OTD‐ and NP‐induced lesions. In both snus groups, we documented the presence of inflamed minor salivary glands adjacent to the snus lesion as yes or no.

### 2.4 Questionnaire

All participants completed a study‐specific questionnaire consisting of a few close‐ended but predominantly multiple‐choice questions. Of note, the questionnaire had not undergone validation. The questionnaire included items on type of snus (NPs, OTD snus, or non‐user), brand and flavour, duration (3–6 months, 6–12 months, 1–2 years, 2–4 years, 4–6 years), frequency (daily, every other day, a few times a week, rarely), quantity (5–10, 11–15, 16–20, > 20 pouches/day), exposure time of each pouch (< 10 min, 11–30 min, 31–60 min, > 1 h), location of the pouch and whether the location varies, subjective oral symptoms, oral hygiene habits, education level, and previous tobacco and nicotine or snus substitute use.

### 2.5 Statistical Analysis

The statistical analyses were performed using R version 4.3.0 (a language and environment for statistical computing and graphics) and RStudio 2023.03.0 (Integrated Development Environment for R).

A non‐inferiority analysis was conducted, testing the null hypothesis that the prevalence of lesions among users of NPs was at least 30% lower than among OTD users. The non‐inferiority margin was chosen to define the maximum clinically acceptable difference in mucosal responses between NP users and OTD users. This threshold was selected based on the known variability and generally benign character of snus‐related mucosal lesions and reflected the level at which a difference would be considered clinically meaningful.

*Proportion of users of NPs with lesions—proportion of OTD snus users with lesions ≤ −0.3*.

*Proportion of users of NPs with lesions—proportion of OTD snus users with lesions > −0.3*.

Based on an initial sample of 33 users of OTD snus and 81 users of NPs, power calculations indicated that a minimum of 39 users of OTD snus and 95 users of NPs would be required to demonstrate non‐inferiority within a 20% margin. Following the recruitment of 63 users of OTD snus and 126 users of NPs, updated calculations showed that an OTD group of 59 users and an NP group of 119 users would be sufficient to establish non‐inferiority within a 30% margin.

We rejected the null hypothesis and concluded that we have at least as many lesions in users of NPs as we have in OTD users with a *p*‐value of 0.00012 when allowing for a margin of 30%. The associated *p*‐value was 0.00012, supporting the conclusion of non‐inferiority. Post hoc analysis of erythema demonstrated that the findings remained robust even when applying a more stringent non‐inferiority margin of 1%. In the univariable analyses, the chi‐square test was used when all expected cell frequencies exceeded 5, and Fisher's exact test when an expected frequency was below 5. For contingency tables with dimensions exceeding 10 × 5, *p*‐values were estimated using the Monte Carlo simulation.

To identify which levels contributed to the observed differences, separate logistic regression models were constructed for each explanatory variable in relation to each dependent variable. For binary outcomes, Firth's bias‐reduced penalised‐likelihood logistic regression was used (the *logistf* package for R). For ordinal outcomes, ordered logistic regression was performed (the *polr* package for R). For each questionnaire‐dependent variable, a multivariable analysis was done with group as the main predictor and age included as a covariate. Binary outcomes were analysed using standard logistic regression (the *glm* package for R), while ordinal outcomes were modelled using the *polr* package. In the tertiary analysis, univariable comparisons between explanatory and outcome variables were performed within the NP user group exclusively. This analysis included explanatory variables such as snus flavour, and outcome variables including tissue reaction and severity scale. Due to the small sample sizes in certain flavour categories, these were aggregated into a single category labelled ‘other flavour’ to ensure statistical robustness.

## 3 Results

Mean age across all groups was 22 years with no significant differences between the groups. While around half of each user group reported a history of smoking, only 10 (12%) of the controls did so. Across all three groups, most former smokers reported having smoked no more than a few times per week and had ceased smoking more than 2 years prior. The difference between the snus user groups was not significant (Table 1).

**TABLE 1:** Study cohort characteristics of the users of nicotine pouches and of oral tobacco‐derived pouches, and of the control group.

	Nicotine pouch users (*n* = 126)	Oral tobacco‐derived pouch users (*n* = 63)	Controls (*n* = 83)
Gender (M/F)	62/64	57/6	35/48
Age ±	22	23	21
History of smoking	66 (54%)	29 (46%)	10 (12%)
Smoking cessation			
3–6 months	10 (9%)	7 (12%)	0 (0%)
6–12 months	13 (11%)	5 (9%)	3 (4%)
12–24 months	19 (17%)	10 (17%)	3 (4%)
> 2 years	48 (42%)	25 (43%)	14 (17%)
Tooth brushing			
< 1/day	4 (3%)	1 (2%)	2 (2%)
1/day	14 (11%)	6 (10%)	5 (6%)
2/day	106 (84%)	55 (87%)	75 (90%)
> 3/day	2 (2%)	1 (2%)	1 (1%)
No answer	0 (0%)	0 (0%)	0 (0%)
Interdental cleaning			
Interdental brush	6 (5%)	7 (11%)	4 (5%)
Dental floss	25 (20%)	16 (25%)	22 (27%)
Plackers	40 (32%)	15 (24%)	28 (34%)
Toothpicks	9 (7%)	2 (3%)	2 (2%)
Highest level of schooling^a^			
High school	70 (57%)	42 (68%)	50 (61%)
Adult Education^b^	7 (6%)	1 (2%)	0 (0%)
Compulsory^c^	8 (70%)	2 (3%)	5 (6%)
University	37 (30%)	17 (27%)	27 (33%)
No answer	4 (3%)	1 (2%)	1 (1%)

^a^ From which a diploma was received.

^b^ Residental College for Adult Education.

^c^ Compulsory education in Sweden: 9 years.

The majority in both groups had used snus for less than the maximum duration of 6 years specified by the inclusion criteria. Among users of NPs, 53 individuals (42%) had used snus for 2–4 years, whereas 37 individuals (59%) in the OTD group reported a snus use duration of 4–6 years. Mixed use of both NP and OTD was rare, with 97% of individuals in both groups reporting exclusive use of one product type. Most users in the NP group rarely changed flavour, but variation of brands within their NP‐snus category occurred. Daily snus use was prevalent in both user groups. Among these, the most commonly reported daily consumption ranged from 5 to 10 pouches. Pouch retention among users of NPs was predominantly reported as approximately 11–30 min, whereas users of OTD snus more frequently reported retentions of 31–60 min. Among users of NPs, more men than women retained the pouch for 31–60 min, whereas no significant gender differences in retention were observed for other durations. Users of both NP and OTD snus usually placed their pouch in the upper labial or buccal sulcus. Approximately the same proportion of individuals in both groups reported varying the location of the pouch (Table 2).

**TABLE 2:** Use characteristics of nicotine pouches and oral tobacco‐derived pouches.

Use characteristics	Nicotine pouches (*n* = 126)	Oral tobacco‐derived pouches (*n* = 63)
Duration of use		
3–6 months	7 (6%)	0 (0%)
6–12 months	14 (11%)	1 (2%)
1–2 years	20 (16%)	6 (10%)
2–4 years	53 (42%)	17 (27%)
4–6 years	30 (24%)	37 (59%)
No answer	2 (2%)	2 (3%)
Mix users		
Between groups	4 (3%)	2 (3%)
Same group	42 (34%)	15 (24%)
Daily use	124 (98%)	63 (100%)
No. of pouches/day		
5–10	58 (46%)	21 (33%)
11–15	42 (34%)	20 (32%)
16–20	24 (19%)	13 (21%)
> 20	1 (1%)	9 (14%)
No answer	1 (1%)	0 (0%)
Exposure duration		
< 10 min	9 (7%)	2 (3%)
11–30 min	57 (45%)	15 (24%)
31–60 min	50 (40%)	40 (63%)
> 1 h	10 (8%)	6 (10%)
Upper labial or buccal sulcus	123 (98%)	63 (100%)
Site variation	56 (44%)	26 (41%)

There was considerable variation in flavour preferences, particularly within the NP group, where a total of 40 distinct flavours were reported. The most preferred NP flavour was ‘Ice Cool’ (*n* = 36, 32%), followed by ‘Freeze’ (*n* = 20, 18%) and ‘Mint’ (*n* = 15, 13%), all of which were mint or peppermint flavoured.

Most individuals in all three groups reported brushing their teeth with fluoride toothpaste twice a day. In general, half of each group reported approximal teeth cleaning.

High school was the most frequently reported highest level of education across all three groups (Table 1).

### 3.1 Oral Symptoms

Fifty‐five individuals in the NP group (44%) and 22 in the OTD group (35%) reported oral symptoms associated with snus. The most frequently reported symptom in both groups was a burning sensation of the oral mucosa: 21 users of NP (17%) and 9 of OTD snus (14%). Five individuals (8%) in the OTD group and 6 (5%) in the NP group reported tooth hypersensitivity, with a similar low frequency in both groups.

### 3.2 Snus‐Induced Lesions

Snus‐induced oral lesions were observed in 56 users of OTD snus (89%: 52 men, 91%; and 4 women, 67%), and in 99 users of NP (79%: 53 men, 85%; and 46 women, 72%). The one‐sided confidence interval for the difference (−0.103 [−0.192, 1]) supported the conclusion of non‐inferiority. Overall, female participants exhibited less pronounced mucosal lesions, whereas male participants tended to present with more severe tissue reactions. No significant association was found between age and prevalence of snus‐induced mucosal lesions in either snus user group. Furthermore, no significant correlation was observed between lesion occurrence and duration of snus exposure. However, the findings suggest that retaining snus in place for 11–60 min may have a greater impact on the oral mucosa compared to durations shorter than 11 min or longer than 1 h (Table 3).

**TABLE 3:** Tissue response in the nicotine pouch group (*n* = 124) according to snus exposure time, graded according to the *Severity Scale*.

Tissue response (grade)	Snus exposure time (min)			
	< 10 (*n* = 9)	11–30 (*n* = 55)	31–60 (*n* = 50)	> 60 (*n* = 10)
0	3 (33%)	12 (22%)	6 (12%)	3 (30%)
1	3 (33%)	27 (49%)	19 (38%)	3 (30%)
2	2 (22%)	14 (25%)	21 (42%)	3 (30%)
3	1 (11%)	2 (4)	4 (8%)	1 (10%)

*Note:* The *Severity Scale*, developed specifically for this study, has four grades: 0 = normal oral mucosa. 1 = mild response severity (solely mild erythema and/or discrete hyperkeratosis). 2 = moderate response severity (moderate erythema and ulceration and/or hyperkeratosis). 3 = severe response severity (severe erythema and ulceration and/or hyperkeratosis).

Snus‐induced lesions among users of OTD exhibited more pronounced hyperkeratosis compared with the lesions observed in users of NPs, with 50 users of OTD (79%) affected versus 82 users of NPs (65%; Table 4, Figure 1). The odds ratio (OR) for hyperkeratosis presence increased with more pouches used (11–15 pouches/day: 2.95; 16–20 pouches/day: 5.21; > 20 pouches/day: 18.5; *p* = 0.00007, 5–10 pouches/day). However, comparing the prevalence of hyperkeratosis in snus lesions between groups, we only observed a tendency toward a difference (*p* = 0.0644). Similarly, the OR for hyperkeratosis thickness increased with the number of pouches used (11–15 pouches/day: 2.3; 16–20 pouches/day: 2.56; > 20 pouches/day: 6.59; *p* = 0.000999). No difference was observed between the groups.

**TABLE 4:** Dental and clinical findings in the oral mucosa among users of nicotine pouches, oral tobacco‐derived pouches, and controls.

	Nicotine pouch users (*n* = 126)	Oral tobacco‐derived pouch users (*n* = 63)	Controls (*n* = 83)
Hyperkeratosis	82 (65%)	50 (79%)	
Mucosal erythema	55 (44%)	20 (32%)	
Oedema	19 (15%)	4 (6%)	
Etch damage	10 (8%)	3 (5%)	
Ulcers	2 (2%)	0 (0%)	
Generalised gingivitis			
None	1 (1%)	2 (3%)	2 (2%)
Discrete	100 (79%)	42 (67%)	76 (92%)
Moderate	20 (16%)	16 (25%)	5 (6%)
Severe	4 (3%)	3 (5%)	0 (0%)
Probing pocket depth ≥ 4 mm in molars and/or incisors	48 (38%)	30 (48%)	24 (29%)
Probing pocket depth ≥ 4 mm in teeth adjacent to snus placement	14 (11%)	11 (18%)	
Bleeding on probing in molars and/or incisors	81 (64%)	42 (67%)	46 (55%)
Bleeding on probing in teeth adjacent to snus placement	58 (46%)	33 (52%)	
Plaque and calculus			
None	3 (2%)	1 (2%)	1 (1%)
Discrete	89 (71%)	42 (67%)	70 (84%)
Moderate	24 (19%)	16 (25%)	11 (13%)
Severe	9 (7%)	4 (6%)	1 (1%)
General gingival recession	57 (45%)	33 (52%)	5 (6%)
Gingival recession in teeth adjacent to snus placement	55 (44%)	33 (52%)	0 (0%)
1–3 mm	50 (40%)	30 (48%)	
4–6 mm	5 (4%)	3 (5%)	
Caries in teeth outside snus placement	22 (17%)	16 (25%)	10 (12%)
Caries in teeth adjacent to snus placement	3 (2%)	2 (3%)	
Osseous defect adjacent to snus placement	0 (0%)	0 (0%)	

**FIGURE 1:**
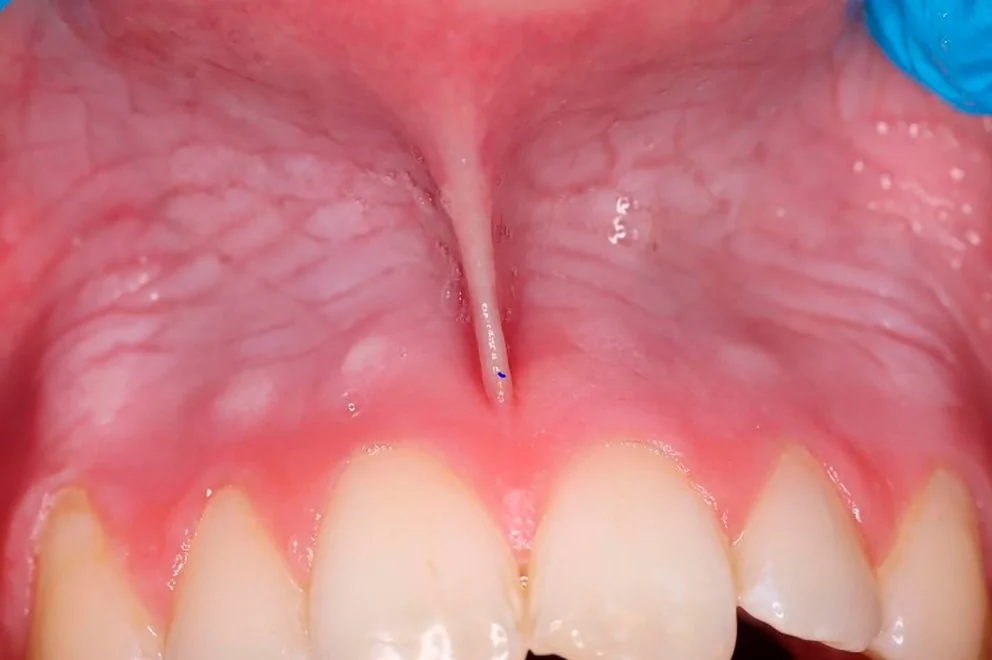
Snus‐induced oral mucosal lesion associated with oral tobacco‐derived snus. The lesion demonstrates epithelial thickening accompanied by surface hyperkeratosis.

Mucosal erythema associated with snus lesions was more frequently observed among users of NPs compared to users of OTD snus. The one‐sided confidence interval for the difference was 0.119 [−0.002, 1], supporting a potential trend toward higher prevalence in the NP group. Consistent with previous findings, the analysis demonstrated that the prevalence of erythema in users of NPs was at least equivalent to that in users of OTD, within a non‐inferiority margin of 30%, *p* = 5.7 × 10^−9^. Oedema was more common in users of NPs; however, it was not a significant difference, and etch damage was infrequent in both groups. Two users of NPs presented with ulcers, whereas no ulcers were observed in the OTD group (Table 4).

Based on the *Tissue Reaction Scale*, the majority of individuals in the NP group were classified as grade 1, while approximately one‐quarter were classified as grade 2 (Table 5). When evaluating snus‐induced lesions using the *Severity Scale*, findings were evenly distributed between grade 1 and grade 2, with 8 cases (6%) classified as grade 3 (Table 6, Figure 2a–d). However, according to the grading criteria of Axell et al., grade 3 lesions, characterised by whitish‐yellowish to brown, wrinkled mucosa with intervening furrows of normal coloration, and marked thickening, were the most frequently observed lesion type in both groups. These were identified in 47 users of NPs (37%) and 33 users of OTD (52%). Inflamed minor salivary glands at the snus site were more common in the NP group (Table 5, Figure 3).

**TABLE 5:** Tissue reaction to snus in the nicotine pouch group (*n* = 126) according to the *Tissue Reaction Scale* and to clinical observation.

Tissue reaction	Users, no. (%)
Tissue Reaction Scale	
Grade 0	24 (19%)
Grade 1	69 (55%)
Grade 2	31 (25%)
Clinical observation	
Inflamed minor salivary glands	21 (17%)
NA	2 (2%)

*Note:* The *Tissue Reaction Scale*, developed specifically for this study, has three grades: 0 = normal oral mucosa. 1 = predominantly hyperkeratosis. 2 = predominantly erythema and/or ulcers.

**TABLE 6:** Gender differences in tissue responses to snus use in the nicotine pouch group (*n* = 126) according to the *Severity Scale*.

Response severity	Nicotine pouch users, no. (%)	
	Female	Male
Severity Scale (grade)		
0 (*n* = 24)	17 (71)	7 (29%)
1 (*n* = 52)	25 (48)	27 (52)
2 (*n* = 40)	19 (48)	21 (53)
3 (*n* = 8)	2 (25)	6 (75)
NA (*n* = 2)	1	1

*Note:* The *Severity Scale*, developed specifically for this study, has four grades: 0 = normal oral mucosa. 1 = mild response severity (solely mild erythema and/or discrete hyperkeratosis). 2 = moderate response severity (moderate erythema and ulceration and/or hyperkeratosis). 3 = severe response severity (severe erythema and ulceration and/or hyperkeratosis).

**FIGURE 2:**
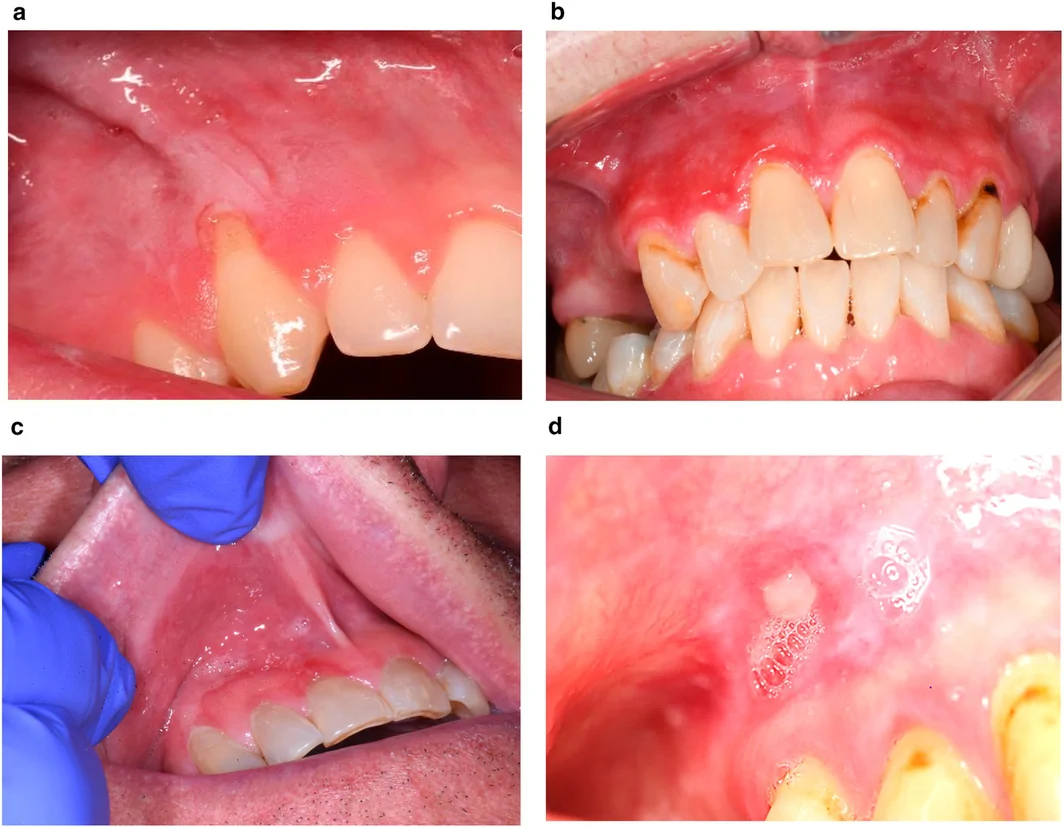
Snus‐induced lesions in the oral mucosa associated with nicotine pouch use (NP) (a) Grade 1 snus‐induced lesion (Severity Scale) in the oral mucosa. The lesion is characterised by mild erythema and discrete hyperkeratosis. (b) Grade 2 snus‐induced lesion (Severity Scale) in the oral mucosa. The lesion displays moderate erythema with early signs of ulceration. (c) Grade 2 snus‐induced lesion (Severity Scale) in the oral mucosa. The lesion presents moderate erythema and early ulcerative changes. Notably, the erythema extends beyond the site of pouch placement, suggesting a broader local inflammatory response. (d) Grade 3 snus‐induced lesion (Severity Scale) in the oral mucosa. The lesion is characterised by a lichenoid reaction, marked erythema, and ulceration.

**FIGURE 3:**
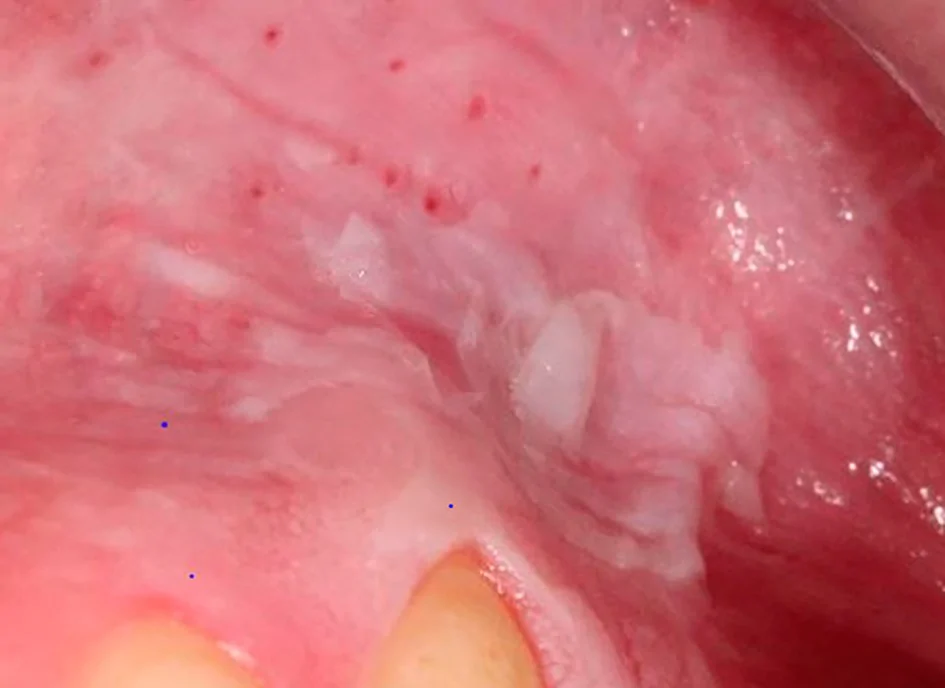
Grade 2–3 snus‐induced lesion (Severity Scale) in the oral mucosa. The lesion is characterised by epithelial desquamation, exposing the underlying connective tissue. Inflamed minor salivary glands are visible as small red dots, indicating localised inflammation.

Among users of NPs presenting with erythema and ulcerations, a correlation with mint‐flavoured snus was observed, although this correlation did not reach statistical significance. A total of 84 users reported a preference for the mint‐flavoured products labelled ‘Freeze’, ‘Ice Cool’, ‘Mint’, ‘Hyperstrong Mint’, and ‘Apple mint.’ Additional mint‐flavoured products were present, although at a lower frequency and sometimes with the flavour concealed behind the product name. According to the Severity Scale, 38 individuals using mint‐flavoured products (45%) exhibited grade 2–3 lesions (Table 7). No significant associations were identified between other flavour variants or specific product characteristics, such as declared nicotine content, moisture level, wrapping, or pouch size, and the presence or severity of snus‐induced oral lesions.

**TABLE 7:** Association between tissue reaction to snus according to the *Severity Scale* and the five most commonly used mint‐flavoured snus in the nicotine pouch group (*n* = 84).

Severity scale (grade)	Most commonly used mint‐flavoured snus, no. (%)				
	Ice cool	Freeze	Mint	Hyperstrong mint	Apple mint
	36 (43)	20 (24)	15 (18)	7 (8)	6 (7)
0 (*n* = 13)	3 (8)	2 (10)	5 (33)	1 (14)	2 (33)
1 (*n* = 33)	19 (53)	6 (30)	6 (40)	1 (14)	1 (17)
2 (*n* = 34)	13 (36)	10 (50)	3 (20)	5 (71)	3 (50)
3 (*n* = 4)	1 (3)	2 (10)	1 (7)	0 (0)	0 (0)

*Note:* The *Severity Scale*, developed specifically for this study, has four grades: 0 = normal oral mucosa. 1 = mild response severity (solely mild erythema and/or discrete hyperkeratosis). 2 = moderate response severity (moderate erythema and ulceration and/or hyperkeratosis). 3 = severe response severity (severe erythema and ulceration and/or hyperkeratosis).

### 3.3 Dental Pathology

Forty‐seven (37%) users of NPs, 30 (48%) of OTD snus, and 24 (29%) controls had a PPD ≥ 4 mm in teeth measured in the incisor and molar region. Individuals with pathological PPD usually had 1–3 affected teeth (NP: *n* = 27, 21%; OTD: *n* = 19, 30%; controls: *n* = 19, 23%). In teeth adjacent to the snus placement site or the snus‐induced lesion, most users in both groups had a PPD of < 4, indicating no apparent correlation between snus use and periodontal pocket formation in these areas (Table 4). Around half of both snus groups exhibited BoP; the difference between groups was not significant. BoP in the snus‐associated region corresponded with BoP levels in the rest of the oral cavity and was consistent across all groups. Caries was uncommon; in all groups, most had no caries (Table 4). Gingival recession at the site of snus placement was observed in around half of each snus user group. Differences between the two snus groups were not significant. No gingival recession was identified in the control group at the corresponding locations (Table 4).

## 4 Discussion

This study explored the oral tissue response, dental health status, and snus habits among young adults using NPs and compared these outcomes with individuals using OTD pouches and a control group of non‐users. Participants were aged 18–30 years, reflecting national patterns of daily snus use in Sweden. Swedish epidemiological data indicate that daily use is most prevalent among individuals aged 16–29 years, with a gradual decline observed with increasing age (Public Health Agency of Sweden 2025).

While smoking prevalence shows marked disparities across population groups defined by education, income, and employment status, such socioeconomic gradients are not observed among snus users, a pattern that was also reflected in our findings (Swedish National Public Health Survey 2025). The findings indicated that female snus users were significantly more likely to use NP products compared to OTD. This increasing prevalence, particularly among younger age groups, is likely to be influenced by multiple factors. These include targeted marketing strategies and product designs tailored to appeal to women, such as flavoured variants and discreet packaging. Furthermore, NPs are often perceived as a cleaner and more socially acceptable alternative to smoking. Changing social norms, declining smoking rates, and the influence of social media trends also appear to contribute to this rise.

Importantly, earlier studies have shown that this trend does not seem to be primarily driven by former smokers transitioning to alternative nicotine products, but rather by a growing uptake among new user groups, especially young women (Swedish Research Council for Health, Working Life and Welfare 2025). In our study, approximately half of the individuals in both snus groups had engaged in occasional social smoking earlier in life. This was more common than in the control group, where only 12% had ever smoked. This suggests that the snus users had a more positive attitude toward tobacco or nicotine products than the control group. Across all groups, the majority of participants ceased smoking more than 2 years prior, thereby minimising the likelihood that previous smoking behaviour influenced the results.

The most common placement site for snus, regardless of type, was the upper labial or buccal sulcus, although other possible sites for snus placement were observed, such as the lower labial or buccal sulcus. Clinical observations indicate that the sublingual area is sometimes used for snus placement, which highlights the importance of a thorough examination of the entire oral cavity. In total, 82% of our study cohort presented with a snus‐induced lesion and 47% with gingival recession in the snus area. This is consistent with the findings of Kopperud et al. who found that 79% of daily 18‐ to 20‐year‐old snus users in Norway had snus‐induced lesions and approximately 20% with gingival recessions (Kopperud et al. 2023). The higher prevalence of gingival recession in the snus placement area observed in our study, particularly among users of OTD, may be attributed to the older age of the individuals, suggesting a longer duration of snus use and, consequently, greater cumulative exposure leading to recession.

The most reported retention duration among users of NPs was approximately 11–30 min whereas users of OTD snus more frequently reported retention times of 31–60 min. These findings diverge from those reported by Liu et al. (2022), who found that Swedish users of NPs tend to use the product for a longer duration (median: 47 min) compared to users in other countries (median: 19 min). They concluded that this difference may reflect Sweden's longstanding cultural tradition of snus use. In our study, the observed differences between users of NPs and OTD products may similarly be influenced by cultural factors, as users of NPs represent a newly emerging subgroup within the broader population of snus consumers.

Gender‐based comparisons, however, revealed that a greater proportion of men retained NPs in place for 31–60 min, and snus‐induced lesions were more prevalent and pronounced among male users. These findings may be attributed to differences in usage patterns, including longer retention times, a preference for stronger flavours, and potentially larger pouch sizes, which may lead to increased local concentrations of active substances and greater mechanical impact on the oral mucosa. The results suggest that an exposure duration between 11 and 60 min may exert a more pronounced effect on oral tissue compared to shorter or longer retention times, likely due to reduced mechanical wear and lower local concentrations in shorter exposures. With longer exposure, the mechanical wear decreases as the snus pouch becomes lubricated by saliva and, by that time, has released all contents.

Maximum blood concentrations of nicotine and total nicotine following use generally increase with both nicotine strength and duration of use (Azzopardi et al. 2025). This supports the hypothesis that the development of snus‐induced lesions is not primarily driven by nicotine itself, but rather by other contributing factors. Additionally, differences in brands may have influenced the outcomes, as it was not uncommon for individuals to switch between various brands within each category.

To date, since no previous studies have defined clinical non‐inferiority for snus, we established a 30% margin based on clinical experience and judgements about what constitutes a meaningful risk difference. Within this predefined margin, NPs cannot be regarded as producing fewer or milder mucosal alterations than OTD. These findings challenge the common assumption that NPs, by being tobacco‐free, are safer for the oral mucosa. To ensure systematic and comparable assessment of snus‐related lesions, we applied standardised grading scales specifically developed for this study. The use of these structured scales enabled consistent evaluation of both the presence and severity of individual lesion components across participants and product types. Snus‐related lesions often present as multifactorial tissue reactions with variable degrees of epithelial thickening, keratinisation, and inflammatory change. A tailored assessment tool was therefore required to distinguish subtle early alterations from more advanced lesion stages.

Even though snus lesions were more common among users of OTD, lesions associated with NPs exhibited greater variability in tissue response patterns. A considerable proportion of users of NPs exhibited hyperkeratosis within the snus‐associated lesions. Many also presented with erythema, while a smaller subset showed ulcerative changes. In contrast, among users of OTD, the majority displayed hyperkeratosis, and no cases of ulceration were observed. The thickening of the hyperkeratotic layer among snus users increased in relation to the number of pouches used, consistent with previous descriptions of hyperkeratinisation as a response to mechanical wear, friction, irritation, and surface etching of the oral mucosa (Nautiyal et al. 2024).

Among users of NPs who did not develop hyperkeratosis, the clinical presentation ranged from mild erythema to lichenoid lesions with ulceration. In some individuals, the erythema extended well beyond the site of pouch placement, indicating a broader inflammatory response. The broader spectrum of tissue reactions observed among NPs users may reflect variations between products within the same category, potentially resulting in more diffuse irritation or heightened inflammatory activation. Snus users often continue product placement despite discomfort. As a result a persistent inflammatory response could contribute to epithelial breakdown and ulceration, increased susceptibility to secondary infections, and the development of chronic irritation if the underlying stimulus remains long term. Persistent inflammation can induce adaptive tissue changes, including hyperkeratosis, acanthosis, and localised fibrosis. When inflammation progresses further, the presence of widespread erythema or more diffuse mucosal involvement may signal a broader inflammatory response rather than a purely localised mechanical effect (Serhan et al. 2010). Repeated mechanical trauma from snus use may also lead to inflammation of the excretory ducts or localised irritation of the minor salivary glands, potentially contributing to the inflammatory responses observed particularly among users of NPs (Hackman 2017). Although OTD‐associated lesions generally resolve following cessation of use, prolonged inflammatory activity sometimes seen with NPs may hinder healing and contribute to recurrent or more complex mucosal alterations.

The differing lesion patterns observed between NP and OTD users suggest that distinct pathogenic mechanisms may underlie these mucosal responses. In OTD users, the predominance of hyperkeratosis is consistent with well‐established pathways driven by chronic mechanical or chemical irritation and repeated friction from pouch placement (Nautiyal et al. 2024). In contrast, the inflammation‐dominated presentation among some of the NP users indicates that factors beyond mechanical trauma may contribute to tissue irritation. Variations in composition, pH, moisture content, wrapping material, or nicotine delivery kinetics may provoke broader inflammatory responses or localised epithelial stress, resulting in the more heterogeneous lesion profiles observed in this group.

Together, these findings demonstrate that the absence of tobacco does not preclude local irritation or tissue alteration and that NPs cannot be assumed to confer a lower risk for oral mucosal changes than OTD. Understanding these divergent lesion profiles is essential, as they likely reflect different mucosal response pathways induced by NPs and OTD snus.

It was evident that mint‐flavoured snus products elicited a more pronounced tissue reaction. When evaluating the supplement facts for the products in question, we found no major differences in content, size, wrapping or moisture level among them. Flavouring agents are incorporated into NP products not only to enhance palatability and provide a cooling sensation, but also to mask the taste of other potentially unpleasant ingredients. In our study, mint‐flavoured products were commonly selected and equally preferred by both men and women. However, men more frequently opted for the ‘Ice‐cool’ variant, which is flavoured with peppermint and menthol, whereas women tended to choose the ‘Mint’ variant, characterised by carvone and spearmint flavouring.

The three additional commonly occurring mint‐flavoured products were ‘Freeze’, which is also characterised by a flavour profile combining peppermint and menthol; ‘Hyperstrong Mint’, whose flavour was formulated using extra strong mint; and ‘Apple Mint’, flavoured with apple, peppermint, and spearmint as indicated by the listed ingredients. Mint flavourings commonly consist of complex mixtures, including peppermint, spearmint, wild mint, and menthol. Additionally, some products contain *Mentha arvensis* (corn/field mint), which is used to enhance the cooling sensation due to its high menthol content. The concentrations of other flavouring agents and other aromatic compounds in Swedish snus products can vary considerably and are often not disclosed by manufacturers.

Transient Receptor Potential Melastatin (TRPM8), also known as the cold and menthol receptor (CMR1), is a temperature‐sensitive ion channel expressed in sensory neurons. It is activated by cool temperatures and compounds such as menthol. Upon activation, TRPM8 allows the influx of calcium and sodium ions, leading to neuronal depolarization and the perception of a cooling sensation. This mechanism explains the cold feeling induced by menthol‐containing products, even in the absence of actual temperature change. The receptor is also activated by peppermint, due to its menthol content (Xu et al. 2020). It is possible that high concentrations of menthol, mint oil and occasionally synthetic cooling agents, all components found in nicotine pouches, may overstimulate this receptor and contribute to irritation and, in some cases, inflammatory responses, particularly in sensitive oral tissues (Kim et al. 2020). Mint may also exert a local irritative or erosive effect on the oral mucosa, which could account for the more pronounced tissue response observed, but flavouring substances are also among the most frequently reported allergens in cosmetic and oral hygiene products, such as toothpastes (Kroona et al. 2017).

The most common adverse reactions to toothpaste include local irritation and contact allergic responses, typically presenting as perioral dermatitis and/or stomatitis, often in the form of oral lichenoid lesions (Kroona et al. 2018; Morton et al. 1995). A retrospective study by Gunatheesan et al. (2012) identified an association between spearmint allergy and the presence of oral lichenoid lesions, suggesting that spearmint may act as a trigger for such reactions. Based on these observations, it is reasonable to hypothesise that the inclusion or concentration of mint‐derived flavouring agents in NP products may reach levels capable of triggering adverse reactions in sensitive individuals. A greater proportion of individuals using NP products also reported symptoms, mostly described as a burning sensation, which correlates with the observed inflammatory response.

The prevalence of caries and periodontitis was generally low and comparable across all three groups. This reflects the current oral health status among 18‐ to 30‐year‐olds in Sweden. The findings are also consistent with several previous studies, in which no association could be demonstrated between snus use and caries, periodontitis, or gingivitis (Wickholm et al. 2004; Rolandsson et al. 2005; Monten et al. 2006; Hugoson and Rolandsson 2011; Hellqvist et al. 2015). It is important to note, however, that these studies only include users of OTD snus. NPs have only been on the market for a short period of time, and prospective epidemiological studies on dental health are currently lacking.

Several methodological limitations must be acknowledged, which may influence the interpretation and generalisability of the results. The study population displayed considerable variation in individual use patterns, yet participants were analysed as a homogeneous cohort. This aggregation limits the ability to detect potential dose–response relationships and the absence of stratification by daily pouch use or years of use further constrains the interpretability of the findings. Furthermore, the descriptive cross‐sectional design provides only a single time‐point snapshot, preventing conclusions about causality or temporal sequence between exposure and mucosal alterations. The design is also susceptible to selection bias, particularly through self‐selection, and to information bias due to reliance on self‐reported data. Confounding cannot be fully accounted for, and only prevalence, not incidence or progression, can be assessed.

## 5 Conclusions

Use of Swedish nicotine pouches is associated with a range of mucosal responses, from hyperkeratosis to lichenoid inflammation, which differ from the predominantly hyperkeratotic reactions observed in users of oral tobacco‐derived snus. Mint‐flavoured variants of nicotine pouches were associated with more pronounced tissue changes compared to nicotine pouches containing alternative flavourings and aromatic compounds; however, not significantly. No increased risk of caries or periodontal disease was observed with nicotine pouches used up to 6 years. Further studies are needed to clarify both local and systemic effects of short‐ and long‐term use of nicotine pouches.

## Author Contributions

**Masoumeh Daneshian:** investigation, validation, visualization, writing – review and editing, project administration, data curation, writing – original draft, formal analysis. **Karin Garming Legert:** conceptualization, writing – original draft, writing – review and editing, methodology, validation, visualization, formal analysis, project administration, data curation, supervision. **Pia Skott:** funding acquisition, writing – review and editing, resources, supervision, software. **Lars Fredriksson:** funding acquisition, writing – review and editing, methodology, resources.

## Funding

This work was supported by Public Dental Service.

## Ethics Statement

Publicly available de‐identified datasets were used, exempting the need for ethics approval per institutional guidelines.

## Consent

The authors have nothing to report.

## Conflicts of Interest

The authors declare no conflicts of interest.

## Supporting information


**Data S1:** STROBE statement—checklist of items that should be included in reports of observational studies.

## Data Availability

All data generated or analysed during this study are included in this published article.
